# Factors Influencing Adherence to Self-Care in Patients with Type 2 Diabetes: A Systematic Literature Review

**DOI:** 10.3390/healthcare14070941

**Published:** 2026-04-03

**Authors:** Ann Velander Karlsson, Therese Petersson, Ulrica Lovén Wickman

**Affiliations:** 1 Department of Region Kronoberg, 352 31 Växjö, Sweden; ann.velander-karlsson@kronoberg.se (A.V.K.); therese.petersson@kronoberg.se (T.P.); 2 Department of Health and Caring Sciences, Linnaeus University, 392 31 Kalmar, Sweden

**Keywords:** adherence, adults, factors, self-care, type 2 diabetes

## Abstract

**Highlights:**

**What are the main findings?**

- Selfcare adherence in type 2 diabetes is shaped by multiple interacting factors, not solely by an individual’s knowledge or motivation.
- Knowledge is important but not sufficient; traditional patient education alone does not consistently lead to improved adherence.

**What are the implications of the main findings?**

- Interventions to improve self-care adherence must be multidimensional, addressing not only patient knowledge but also psychological, social, economic, and organizational factors.
- Healthcare delivery should prioritize individualized and psychologically informed care, ensuring that patients experience self-care as meaningful, manageable, and socially supported.

**Abstract:**

**Background:** Living with type 2 diabetes can be demanding from multiple perspectives. Many individuals with type 2 diabetes experience limited self-care ability and poor adherence to self-care recommendations. **Objective:** To describe factors that influence self-care adherence among adults with type 2 diabetes. **Methods:** A systematic review based on 19 studies using quantitative, qualitative, and mixed-method designs. The study design is a systematic literature review in accordance with the guidelines of the Swedish Agency for Health Technology Assessment and Assessment of Social Services (SBU) and PRISMA guidelines. The articles were retrieved from the research databases CINAHL, PubMed, and PsycInfo. Quality appraisal was conducted using the MMAT, and the studies were analyzed through meta-aggregation and subsequently compiled into a unified set of findings. **Results:** The findings showed that numerous factors influence adherence to self-care. These were presented in categories: sociodemographic factors; disease- and treatment-related factors; healthcare-related factors; and psychological factors. Psychological factors played a decisive role in adherence, particularly regarding motivation. **Conclusions:** Living with Type 2 diabetes demands considerable engagement and supporting them as a district nurse is a multidimensional task consisting of several components. Self-care adherence in type 2 diabetes cannot be understood as the result of isolated factors but rather as a complex interplay of psychological, social, economic circumstances. Understanding effective patient education enhances knowledge and understanding of the importance of adhering to treatment is the key to improving compliance in patients with type 2 diabetes.

## 1. Background

Type 2 diabetes is a chronic metabolic disease marked by elevated blood glucose due to insulin resistance and a gradual decline in insulin secretion [1,2]. Its development reflects an interaction between genetic predisposition and lifestyle factors such as overweight, diet, physical activity, smoking, and alcohol use [3,4,5]. Although both heredity and unhealthy habits increase the risk, a healthy lifestyle can mitigate it even in genetically susceptible individuals [6,7,8]. Insulin is essential for glucose uptake and normal metabolic function [9], primarily enabling glucose absorption in skeletal muscle and adipose tissue [10,11]. In type 2 diabetes, impaired insulin secretion and reduced insulin sensitivity result in insulin resistance [12,13]. Initially, increased insulin production helps maintain glucose balance [14], but secretion typically declines as the disease progresses [12,13]. Long-standing dysregulation can lead to vascular and neurological complications [13]. Type 2 diabetes develops gradually and mainly affects adults over 50, although early-onset cases are rising among adolescents and young adults [15]. Individuals diagnosed before age 30 may lose an average of 15 years of life expectancy [15]. Living with lifelong conditions like diabetes requires acceptance, knowledge acquisition, and the integration of the disease into daily routines [16]. Effective management involves ongoing monitoring, applying new knowledge, and adjusting self-care and medication to changing circumstances, which influences emotional well-being and personal agency [17]. Routines can provide stability, whereas limited resources and insufficient support increase vulnerability and hinder self-care [16,17].

Individuals with type 2 diabetes are at risk for both short- and long-term complications [2]. Cardiovascular disease is the most common, including increased risk of myocardial infarction, stroke, and hypertension. Diabetic retinopathy can damage retinal blood vessels, potentially leading to vision loss or blindness. Other complications include diabetic nephropathy, which may progress to kidney failure and require dialysis, and diabetic neuropathy, which can cause pain, numbness, and sensory loss, particularly in the feet [2]. Poor circulation related to type 2 diabetes also increases the risk of non-healing wounds and, in severe cases, amputation [16]. Living with type 2 diabetes can be psychologically taxing, as the extensive self-care demands increase the risk of depression and anxiety. Daily attention to diet, physical activity, and monitoring require sustained effort. Maintaining good glycemic control through lifestyle changes and medication can reduce the risk of complications and improve quality of life [17].

Type 2 diabetes can be both prevented and managed through lifestyle changes [18]. Treatment aims to slow disease progression and reduce the risk of complications. It typically includes regular monitoring, medication, and behavioral modifications, with lifestyle habits playing a central role [19]. Key lifestyle factors include diet, physical activity, tobacco use, and alcohol consumption, and optimizing these behaviors is crucial for effective disease control. Improved habits reduce the risk of complications, especially cardiovascular disease [4].

Diet is an essential component of treatment. Diets high in fast-acting carbohydrates cause rapid blood glucose spikes, whereas balanced nutrition supports glycemic control, weight stability, and vascular health [20]. Maintaining a healthy weight is also important, as overweight and obesity impair glucose metabolism and increase complication risk. Physical activity helps prevent overweight and enhances cellular glucose uptake, contributing to metabolic control [21,22,23]. Increased activity has been shown to be particularly effective [24], and daily movement combining aerobic and strength-focused exercise is recommended. Even small increases in activity are beneficial [24,25]. Smoking worsens metabolic control [26], because tobacco reduces insulin sensitivity [27], making smoking cessation an important part of treatment. A comprehensive lifestyle approach with healthy diet, regular physical activity, and weight management promotes metabolic control, reduces medication needs, and lowers cardio-vascular risk [28]. An individual’s self-care capacity and treatment adherence are critical for maintaining metabolic control [19].

Pharmacological treatment includes medications that increase insulin secretion, enhance insulin sensitivity, or reduce glucose absorption into the bloodstream [29]. Treatment must be individualized based on factors such as cardiovascular disease, kidney function, hypoglycemia risk, and body weight [30]. With longer disease duration, insulin therapy often becomes necessary as endogenous insulin production declines [27].

Adherence to self-care refers to how well an individual’s daily behaviors align with recommended treatment plans and self-care strategies. It is a dynamic process in which the person actively participates by initiating, performing, and maintaining recommended routines [31,32]. Adherence reflects how effectively agreed-upon actions are carried out, and high adherence is essential for achieving treatment goals, preventing complications, and improving quality of life. Viewing adherence as a partnership shifts the focus from passive compliance to shared responsibility, emphasizing the individual’s values and resources and strengthening their empowerment [33]. For specialist nurses such as district nurses, understanding the concept of adherence is crucial for supporting effective self-care behaviors [32]. Adherence to self-care is generally low among individuals with type 2 diabetes, contributing to a higher incidence of diabetes-related complications [34].

Type 2 diabetes is a widespread condition that contributes to significant human suffering and substantial societal costs. Living with the disease can be demanding, particularly due to the lifelong self-care burden that requires continuous motivation and engagement. Managing diabetes poses challenges not only for individuals but also for district nurses, especially when patients struggle with low motivation or poor adherence to self-care. Previous research shows that insufficient self-care ability and poor adherence can lead to negative health outcomes and increased suffering. Although multiple factors are known to influence adherence, comprehensive research syntheses in this area are limited. Summarizing the existing evidence is therefore valuable for gaining broader insight into these influencing factors. The results will be discussed in relation to Riegel’s self-care theory [35,36]. which helps clarify how various factors interact and supports deeper understanding. This knowledge can contribute to improvements in diabetes care, particularly for district nurses who support individuals with type 2 diabetes in achieving health, well-being, and effective disease management. By developing a deeper understanding of adherence, healthcare professionals can better recognize, consider, and integrate influencing factors into care. The objective in this study was to describe factors that influence adherence to self-care among adults with type 2 diabetes.

## 2. Materials and Methods

### 2.1. Design

The study design is a systematic literature review. This approach was selected to provide insight into the existing body of knowledge on the subject. The systematic review in this study was conducted in accordance with the procedures outlined [37]. A systematic literature review is conducted to compile current empirical evidence based on a specific research question. A well-executed systematic review enables readers to evaluate the reliability of the study’s conclusions, as it synthesizes available research within a defined area. This systematic review was not registered in advance of conducting the literature search.

### 2.2. Datacollection

#### 2.2.1. Search Strategy

To generate search terms and identify relevant literature aligned with the aim of the study, the PEO model (Population, Exposure, Outcome) was used [37]. The PEO model supported the structuring of the information search by extracting key meaning units based on the study’s purpose. Using the PEO model, relevant free-text terms were identified, and appropriate index terms were retrieved through MeSH (Table 1).

Based on the PEO model, relevant free-text terms were identified, and appropriate indexed terms using MeSH. A final search was conducted in CINAHL, PubMed and PsycInfo. The selected databases are considered appropriate for searches within the field of nursing. Free-text terms and indexed terms were grouped using OR and then combined into a block search using AND to align with the aim of the study. It is essential to formulate a clear research question before initiating a literature search. To narrow the search, inclusion and exclusion criteria were established [37].

Inclusion criteria required that articles were peer-reviewed, published in English, aligned with the aim of the review, and ethically approved. Exclusion criteria specified that studies older than eight years and review articles were to be omitted.

An initial test search was conducted in CINAHL to assess the availability of scientific literature relevant to a systematic review. The final data collection was then carried out through searches in PubMed, CINAHL, and PsycInfo. These databases were deemed appropriate for research within nursing [37]. Data collection was performed in September, 2025. Relevant index terms were retrieved from each database’s subject headings: CINAHL Headings in CINAHL, MeSH in PubMed, and Thesaurus in PsycInfo. Index terms and free-text terms consisting of more than one word were placed in quotation marks to ensure all terms were included in the search. These terms included: “Type 2 diabetes,” “Diabetes type 2,” “Self care,” “Self administration,” “Activit* of daily living,” and “Diabetes mellitus type 2.” Additional terms were “treatment adherence” and “treatment compliance.” Truncation was applied to activity as “activit* of daily living” to capture all word endings [37]. Free-text terms and index terms were combined with the Boolean operator OR into three thematic groups, which were then merged into a block search using AND to match the study aim (Supplementary Materials S1). Articles not peer-reviewed were excluded in CINAHL and PsycInfo using database filters. Because PubMed lacks this function, articles from this database were manually reviewed for quality by both authors. The final search yielded 330 articles. Relevance and quality were assessed in four steps, with exclusions made at each stage.

Step 1: All titles (n = 330) were screened, resulting in 255 exclusions due to duplicates, lack of relevance, or unsuitable study design.

Step 2: Abstracts of the remaining 75 articles were reviewed, and 42 were excluded due to irrelevance, duplication, or other reasons. Duplicates were removed whenever detected.

Step 3: Full texts of 33 articles were read, and 11 were excluded for targeting the wrong population, unclear methodology or structure, lacking ethical considerations, or not aligning with the aim.

Step 4: The remaining 22 articles proceeded to quality appraisal.

#### 2.2.2. Quality Appraisal

Because the included studies applied qualitative, quantitative, and mixed-methods designs, MMAT was selected to allow for a uniform appraisal tool across all articles. The tool consists of five main sections with design-specific criteria, along with two general questions evaluated regardless of study design. Each criterion was answered with “yes,” “no,” “can’t tell,” or accompanied by comments. Studies meeting all quality criteria were rated as high quality; those with one item marked “no,” “can’t tell,” or with comments were rated as moderate quality; and studies with two or more such remarks were considered low quality. Only studies of high or moderate quality were included in the review. Three articles were excluded due to low quality, leaving 19 articles for the final synthesis. The results of the quality appraisal are shown (Table 2, Supplementary Materials S1).

Quality appraisal was conducted for the remaining 22 articles using the Mixed Methods Appraisal Tool (MMAT) [38] (Supplementary Materials S2). Because the included studies employed qualitative, quantitative, and mixed-methods designs, MMAT was selected to ensure a uniform appraisal approach across all articles. The tool comprises five main sections with design-specific criteria, in addition to two general questions assessed regardless of study design. Each criterion was evaluated using the response options “yes,” “no,” “can’t tell,” or accompanied by explanatory comments.

Studies that met all quality criteria were rated as high quality. Those with one criterion rated as “no,” “can’t tell,” or accompanied by comments were classified as moderate quality, whereas studies with two or more such assessments were considered low quality. Only articles assessed as high or moderate quality were included in the review. Three articles were excluded due to low quality, leaving a total of 19 articles for the final synthesis. The results of the quality appraisal are presented in Table 2 and explained in Supplementary Materials S2.

**Table 2:** Article matrix.

Author/s/Title Year, Country	Objective	Method	Quality Score
1. Caner E, Kavuran E [39] Evaluation of the relationship between self-care and treatment compliance in patients with type 2 diabetes: A cross-sectional study from Northeast Türkiye Turkey	The study aimed to investigate the connection between self-care behaviors and treatment adherence among individuals with type 2 diabetes.	A cross-sectional correlational study involving 191 patients	High
2. Cengiz, D, Korkmaz [40] Effectiveness of a nurse-led personalized patient engagement program to promote type 2 diabetes self-management: A randomized controlled trial. Turkey	This study aims to adapt and evaluate the effectiveness of PHEinAction^®^ on diabetes self-management (DSM) among Turkish type 2 diabetes mellitus patients.	A randomized controlled trial	High
3. Endale et al. [41] Adherence to diabetic self-care management and associated factors among type 2 diabetic patients in North Shewa Zone public hospitals in Amhara Region, Ethiopia. Ethiopia.	To assess adherence to diabetic self-care management and associated factors among type 2 diabetic patients in North Shewa Zone public hospitals, Ethiopia, 2023.	Mixed-methods approach	High
4. Gomez Aguilar et al. [42] Type 2 Diabetes Self-Care in Oxcutzcab, Yucatan, Mexico: The Role of Mental Health. Mexico	To understand differences in adherence to type 2 diabetes treatment among individuals who report anxiety, anger, and depression.	A Cross-sectional study	High
5. Hegde et al. [43] Influence of Social Support on Treatment Adherence and Self-care among Type 2 diabetes mellitus patients in Field Practice Areas of a Tertiary Medical College in Bangalore. India	To evaluate the levels of social support in type 2 Diabetes Mellitus (T2DM) patients and to identify the influence of social support on treatment adherence and self-care activities	A Cross-sectional Study.	High
6. Ibrahim et al. [44] Tailoring nursing interventions to empower patients: personal coping strategies and self-management in type 2 diabetes care. Egypt	This study evaluates the effectiveness of a structured diabetes self-management education program on patients’ self-management behaviors, empowerment, and activation levels.	A quasi- experimental design	High
7. Lins Oliveira Frazão et al. [45]. Correlation between symptoms of depression, attitude, and self-care in elderly with type 2 diabetes Brasil	To correlate depressive symptoms, attitude, and self-care of elderly people with type 2 diabetes.	A Cross-sectional Study.	High
8. Mendoza-Catalán et al. [46]. Personality Traits and Self-Care Behaviors in Adults with Type 2 Diabetes Mellitus. Mexico	The purpose of this work was to explore the relationship of personality traits with self-care in Mexican adults with type 2 diabetes mellitus.	A cross-sectional study	High
9. Portela, et al. [47]. Diabetes mellitus type 2: factors related to adherence to self-care. Portugal	To analyze the sociodemographic and clinical variables related to the adherence to self-care activities in people with diabetes mellitus type 2.	A cross-sectional study	High
10. Ramírez-Morros et al. [48]. Impact of Gender on Patient Experiences of Self-Management in Type 2 Diabetes Spain	To identify gender disparities in knowledge, attitudes and behaviors related to self-management and control of Type 2 diabetes Mellitus (T2DM) among primary care patients.	A Qualitative Study	High
11. Rana D, Kumar R, Kant R [49]. Psychological Predictors of Adherence to Self-Care Behaviour amongst Patients with Type 2 Diabetes Mellitus (T2DM) Visiting Public Hospital, North India India	To identify the psychological predictors of self-care behaviors among patients with T2DM.	A descriptive cross-sectional survey	High
12. Sabri et al. [50]. The association between self-care activities and depression in adult patients with type 2 diabetes in Taif City Saudi Arabia	The objective of the study was to examine the association between depression and diabetes self-care activities among adults with type 2 diabetes in Taif City, Saudi Arabia	A cross sectional Study	High
13. Seah et al. [51]. Effectiveness of a cluster randomized controlled trial involving community-based intervention for older adults with type 2 diabetes mellitus in Singapore. Singapore	The purpose of the study was to investigate the effectiveness of a community-based intervention in improving diabetes knowledge, self-care behaviors, and glycemic control among older adults with type 2 diabetes mellitus in Singapore.	A cluster randomized controlled trial	High
14. Silva-Tinoco et al. [52]. Adherence to antidiabetic treatment in primary health care in individuals with type 2 diabetes. A survey including socio-demographic, patient related and clinical factors Mexico	To investigate patient-related factors associated with adherence to antidiabetic treatment in patients with type 2 diabetes in primary health-care units.	A cross-sectional study	High
15. Titilayo O, Modupe A. [53]. Self-care related knowledge and self-care practices among type 2 diabetic patients attending selected hospitals in Oyo State, Nigeria. Nigeria	This study assessed self-care related knowledge and practices among Type 2 Diabetic patients attending selected hospitals in Oyo State, Nigeria	A descriptive cross-sectional design	High
16. Tuobenyiere et al. [54]. Patient perspective on barriers in type 2 diabetes self-management Ghana	To explore the perceived barriers in Type 2 Diabetes care among patients with diabetes.	A qualitative Study	High
17. Wong et al. [55]. Identifying barriers and facilitators to self-care in young adults with type 2 diabetes Singapore	To identify the main barriers and facilitators of self-care behaviours in this population.	A qualitative study	High
18. Zewdie et al. [56]. Self-Care Practice and Associated Factors Among Patients with Type 2 Diabetes Mellitus at a Referral Hospital in Northern Ethiopia Ethiopia	This study assessed the self-care practice and associated factors among type 2 diabetes patients attending their treatment at Dessie Referral Hospital, Dessie, North-Eastern Ethiopia.	A mixed methods design	High
19. Zhu X et al. [57]. The short- and long-term effects of community-family-doctor-based type 2 diabetes self-management interventions. China	To evaluate the short- and long-term effects of community- family-doctor-based self-management interventions for T2DM and to explore strategies for long-term glycemic control.	A randomized controlled trial.	High

#### 2.2.3. Data Analysis

The collected material was analyzed using meta-aggregation, a qualitative synthesis method used to summarize findings and recommendations from multiple studies on a specific topic. Meta-aggregation is a text-close analytical approach that focuses strictly on the results of the primary studies, meaning that no interpretive or theory-driven analysis is performed. All selected articles were carefully read by both authors to gain a deeper understanding of their content. The meta-aggregation followed three steps as outlined [37]. In *the first step*, key findings related to the study aim were extracted from the results sections of each article. The articles were printed in full text and numbered from 1 to 19, and the extracted findings were cut out as paper strips and labeled with the corresponding article number; for example, “Better interaction between patients and healthcare staff to resolve doubts/questions may improve adherence” [53]. In *the second step*, the findings were aggregated by sorting them into groups based on similarities in meaning. Strips with similar content were placed together, forming preliminary categories, and findings belonging to each category were compiled on a shared paper sheet to clarify the emerging structure. An example of a category at this stage was care relationships. In *the third step*, the content of these categories was further examined to identify similarities and differences. The findings were then synthesized into broader, overarching categories, which formed the basis of the systematic review’s results and discussions were performed until consensus [37] (Table 3). Qualitative studies were extracted as meaning-bearing units and preliminary thematic statements. Quantitative results relevant to the review question were synthesized narratively, as recommended by SBU when data are heterogeneous and not suitable for statistical pooling. To enable integration, these quantitative results were transformed into concise textual summaries that preserved the essential findings. Mixed-methods studies were separated into their qualitative and quantitative components prior to extraction [37].

#### 2.2.4. Ethical Considerations

The ethical considerations in this study were guided by the Belmont Report’s principles of respect for persons, beneficence, and justice. Respect for persons involves presenting previous research accurately and with integrity [58]. In accordance with the Declaration of Helsinki [59], the included studies were reviewed for essential ethical components such as anonymity, informed consent, adequate participant information, and evidence of ethical approval.

## 3. Results

**Figure 1:**
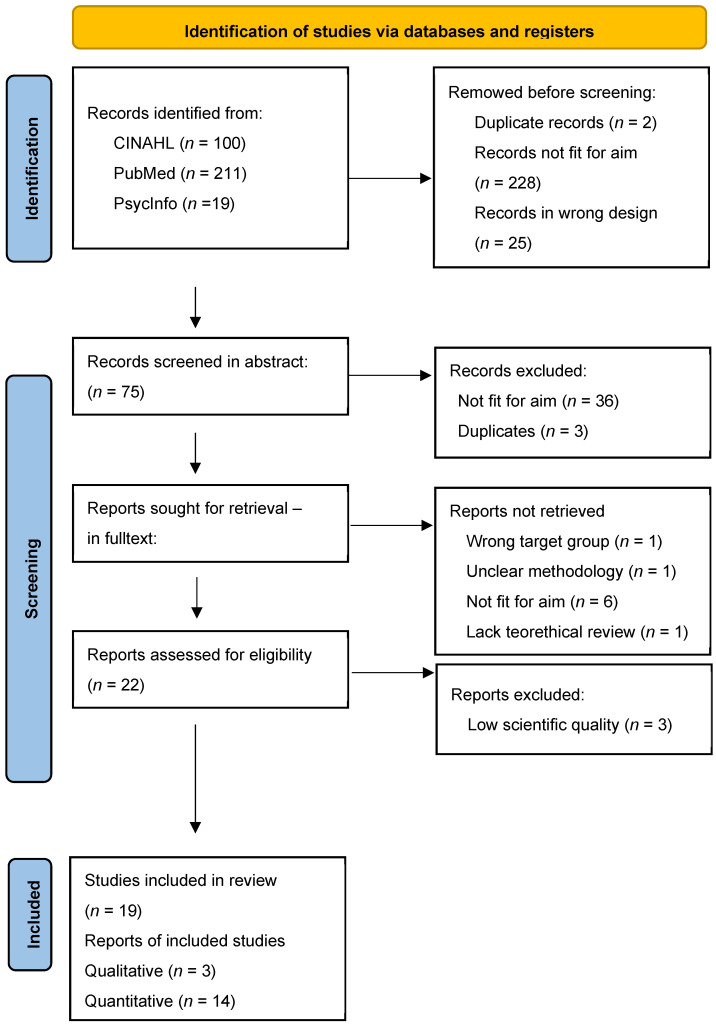
PRISMA flowchart.

The 19 included articles were published between 2022 and 2025 and were conducted in various countries across the world: Ethiopia (n = 2), Turkey (n = 2), Mexico (n = 3), India (n = 2), Egypt (n = 1), Iran (n = 1), Brazil (n = 1), Spain (n = 1), Portugal (n = 1), Singapore (n = 2), Nigeria (n = 1), Ghana (n = 1), and China (n = 1) (Table 2). The results are presented in four main categories that emerged during the meta-aggregation process. To clarify the search process and the structure of each category, including subcategories, see Figure 1 and Table 4.

### 3.1. Sociodemographic Factors

#### 3.1.1. Age

The results show that age influences adherence to self-care [41,45,47,56], although the findings are not consistent. According to Zewdie et al. [56], adherence decreases with increasing age; individuals over 70 were less likely to follow recommendations regarding diet, physical activity, and blood glucose monitoring, partly due to physical limitations, fatigue, and memory problems. Similar findings were reported by Lins Oliveira Frazão et al. [45], where 93% of participants over 60 demonstrated poorer adherence and more negative attitudes toward self-care. In contrast, Endale et al. [41] found the highest adherence among individuals aged 61–70, while those over 70 showed lower adherence than those aged 30–60.

#### 3.1.2. Sex

Two studies identified sex as a factor influencing adherence [41,48]. Ramirez-Morros et al. [48] found that women tended to engage more in self-care; they were more open about their diagnosis, sought emotional support, and attended group-based education. Men focused more on practical tasks such as glucose monitoring and were more reluctant to discuss their diabetes, making dietary adherence in social contexts more difficult. Endale et al. [41] also reported slightly better adherence among women, although the difference was not statistically significant [41]. Women’s tendency to prioritize family needs was suggested as a possible explanation for occasionally reduced self-care [48].

#### 3.1.3. Knowledge- and Work-Related Factors

Knowledge was identified as a factor influencing adherence [41,48,50,52,53,54,55,56]. However, having adequate knowledge about diabetes and self-care did not necessarily lead to improved adherence to self-care behaviors. One study showed that most participants had adequate knowledge yet did not apply it consistently [53]. Sabri et al. [50] found that although 78% had good knowledge, 94% lacked adequate self-care routines. Silva-Tinoco et al. [52] noted that knowledge distinguished adherent from non-adherent individuals, although knowledge alone could not fully explain adherence to self-care [52]. The result emphasized that knowledge must be paired with access to resources and support [41,56]. Attitudes, social roles, and practical conditions shaped how knowledge was used. Accurate knowledge increased confidence and improved self-care, whereas insufficient knowledge created uncertainty [48].

Education and differences in educational attainment influence adherence to self-care [39,41,47,56]. Educational level affected how well individuals adhered to their self-care routines. Individuals with limited formal education had greater difficulty maintaining continuity in their self-care activities [41]. Those with low levels of formal education struggled to interpret blood glucose values, read medication and dietary instructions, and understand complex treatment recommendations, which resulted in poorer continuity, particularly regarding diet and physical activity [47,56]. They often experienced challenges in comprehending and applying the guidance provided by healthcare professionals, leading to reduced adherence due to uncertainty and misunderstandings about the disease [39,47,56]. Individuals with upper-secondary education or higher showed better adherence to recommendations compared with those with little or no education [39]. They were more successful in maintaining recommended dietary habits, physical activity, and regular blood glucose monitoring [47]. Those with higher education were nearly three times more likely to achieve good self-care compared to individuals with only primary education [41]. Individuals who had completed college or university demonstrated the highest levels of adherence; those with higher education had five times better adherence to self-care than individuals without schooling or with only primary education [41].

Employment status and the type of occupation an individual holds influence adherence to self-care [39,41,47,52]. Individuals employed in the public sector, farmers, and those with stable employment demonstrated better adherence to self-care compared with those who were unemployed or had temporary work [41]. Both employment status and occupational type play a role in self-care adherence among individuals with type 2 diabetes [39]. Unemployed individuals and those in lower-skilled occupations generally exhibited poorer adherence compared with those in more stable or skilled professions [39]. Lower adherence among unemployed participants, reflected in reduced engagement in most self-care activities, whereas those with permanent employment followed their self-care routines more consistently [41,47]. Unemployment was a negative predictor of adherence, with unemployed individuals being 66% less likely to adhere to self-care routines compared with those who were employed [41]. These findings contrast with Silva-Tinoco et al. [52] who reported a positive association between unemployment and self-care, as unemployed participants in their study engaged in more self-care activities than those who were employed [52].

#### 3.1.4. Economy and Income Level

The results indicate that financial circumstances influence adherence to self-care [41,47,54]. Individuals without employment could not afford appropriate and recommended diabetes-friendly foods due to limited financial resources [41]. Individuals with higher incomes could exhibit poorer self-care adherence, as they tended to consume more sweets than those with fewer financial means, negatively affecting their self-care behaviors [47]. Participants with limited economic resources reported difficulties affording medications, purchasing blood glucose meters and test strips, and paying for healthy foods. They described how low income or lack of financial resources reduced their ability to carry out recommended self-care routines, resulting in decreased adherence, particularly regarding medication use, self-monitoring, and dietary practices. Financial hardship also contributed to psychosocial stress and the prioritization of other basic needs over diabetes care, which further undermined self-care behaviors [54].

#### 3.1.5. Social Support

Social support is a key factor influencing adherence to self-care in individuals with type 2 diabetes [43,54,55,56]. Lack of social or family support emerged as the greatest barrier to engaging in self-care. Even basic forms of support were considered important, such as verbal reminders to take medication, assistance with purchasing medications, and family involvement in daily self-care activities. When such support was lacking, adherence to self-care declined [56]. Similarly, social support as a critical determinant of self-care adherence in type 2 diabetes, emphasizing that having an established social network was essential for receiving support. Such networks may include close relatives, spouses, siblings, colleagues, or friends [54]. Not only the presence of social support but also its level influenced self-care adherence. Individuals with a high level of social support demonstrated better adherence than those with low support, particularly in relation to physical activity, diet, and foot care. Those with moderate levels of social support showed better adherence to medication regimens and blood glucose monitoring [43]. Furthermore, the result reported that individuals receiving a high degree of social support from friends and family found it easier to maintain lifestyle changes over time, indicating that adherence to self-care improved even in the long term. Concrete forms of support, such as friends or family joining individuals in their lifestyle changes, exercising together or eating healthy meals, were shown to positively influence self-care behaviors. The study underscored the importance of social support in achieving sustainable lifestyle modifications among individuals with type 2 diabetes [55].

#### 3.1.6. Religion

The findings indicate that religion can influence adherence to self-care [55]. Adherence tends to decline when individuals transfer personal responsibility for their health to a higher power. Even when adherence to self-care is generally good, adverse events may still occur, and for religious individuals such events may be attributed to divine intervention or perceived as part of God’s will. This reflects a fatalistic explanatory framework in which events are understood as determined by a divine will, thereby limiting an individual’s perceived control over their health. Under such fatalistic beliefs, personal efforts may be viewed as futile, as they are thought to be overridden by a higher power, which in turn undermines individuals’ sense of responsibility and their expectations that self-care will lead to improvement [55].

#### 3.1.7. Geographical Differences

Adherence to self-care is influenced by an individual’s place of residence [41,54,56]. Individuals living in urban areas tend to demonstrate better adherence to diabetes self-care [54]. Their adherence was more than five times higher than that of individuals residing in rural settings [41]. Challenges for individuals living in rural areas were related to both geography and lifestyle factors [56]. Limited access to diabetes supplies, exercise facilities, and support for lifestyle modification made adherence to dietary recommendations, physical activity, and regular follow-up more difficult for rural residents [41,56]. Long travel distances, high transportation costs, and long working hours further contributed to missed appointments, check-ups, and educational sessions, which negatively affected adherence to self-care [41,54,56].

### 3.2. Disease- and Treatment-Related Factors

#### 3.2.1. Disease-Related Factors and Comorbidity

The findings highlight that several disease-related factors influence adherence to self-care among individuals with type 2 diabetes. These factors include comorbidities, the burden of complications, illness perceptions, and the duration of the disease [41,50,52,55,56]. The result demonstrated that diabetes-related complications negatively affect adherence to self-care, particularly regarding engagement in physical activity [41,56]. Visual impairment and pain were contributing barriers, limiting individuals’ ability to exercise or perform daily self-management tasks. Accordingly, individuals with fewer or no complications exhibited better adherence to self-care [41]. Other studies emphasized that comorbidities may hinder adherence, as coexisting health conditions typically increase the overall disease burden [50,56]. Comorbid kidney disease, for instance, could lead to nausea during physical activity, resulting in reduced participation and diminished adherence to recommended self-care behaviors [56]. Beyond these factors, an absence of diabetes-related symptoms may also negatively influence adherence. When individuals do not experience symptoms, motivation for lifestyle changes and self-care behaviors tend to decline [52]. Diabetes duration plays a role, individuals who had a shorter disease duration displaying better adherence [41]. Finally, exposure to health threats or personal loss often triggered a shift in individuals’ mindset, which in turn promoted increased engagement in self-care activities. Such experiences appeared to strengthen motivation and thereby enhance long-term adherence to self-care [55].

#### 3.2.2. Medication Treatment

The result demonstrated that medication-related factors can influence adherence to the pharmacological component of diabetes management [52]. The study highlighted the occurrence of side effects as a reason for poor adherence, as individuals experiencing adverse effects were less likely to follow their prescribed medication regimen. The authors also emphasized that the number of medications could affect adherence: individuals taking multiple medications tended to be less adherent, whereas those with fewer medications demonstrated better adherence to their treatment. Furthermore, the study reported that individuals who perceived medication intake as disruptive to their daily social activities showed lower adherence to their medical treatment compared with others [52].

### 3.3. Healthcare-Related Factors

#### 3.3.1. Healthcare Organization

The results indicate that the organization of healthcare can be a significant factor influencing adherence to self-care among individuals with type 2 diabetes. Providing individually tailored care as a complement to traditional diabetes care was shown to be effective in improving adherence [40,44,51,57]. Across all studies, individuals who received only traditional diabetes care demonstrated poorer adherence compared with those who received traditional care combined with an individualized intervention [40,44,51,57]. In the study by Cengiz and Korkmaz [40], a nurse-led intervention with a personalized care program was tested. The intervention aimed to enhance patient engagement among individuals in the intervention group. The control group received traditional diabetes care, while the intervention group received both traditional care and the nurse-led intervention. The intervention included two in-person sessions with a nurse at four-week intervals, as well as a telephone consultation between sessions. In addition, participants completed various home-based tasks addressing cognitive, emotional, and behavioral aspects of self-care. At follow-up, the intervention group showed significant improvements in treatment adherence, patient engagement, and self-efficacy. These positive outcomes were attributed to the individualized approach of the intervention, the increased interaction between the nurse and participants, and the inclusion of cognitive, emotional, and behavioral components [40]. Another factor contributing to improved adherence was the incorporation of personalized treatment goals, which positively influenced self-care behaviors. Such goals were found to help individuals maintain focus and direction in their self-management efforts [40,44,55].

#### 3.3.2. Care Relationships

The result indicates that interaction, relationships, and communication with healthcare providers are factors that can influence adherence to self-care [48,52,55,57]. Individuals who were non-adherent to self-care reported dissatisfaction with communication with their physician. Conversely, individuals who experienced good interaction with their healthcare providers demonstrated stronger adherence [57]. This finding was further supported by evidence showing that participants viewed effective communication with healthcare providers as essential for successful self-management and treatment adherence [48,55].

#### 3.3.3. Medical Equipment and Technology

The result highlight a factor that appears to facilitate adherence to self-care, namely having a personal glucometer, Access to glucose meters improved awareness and motivation [51,54]. In a study, participants in the intervention group were provided with free access to a glucometer, lancets, and test strips, which enabled them to monitor their blood glucose both for learning purposes and in response to changes in their health status. These measurements and values served as motivation for making behavioral changes. Participants reported that gaining awareness of their glucose levels increased their motivation to perform and direct their self-care efforts more purposefully [51]. In contrast, no significant improvement in self-care among individuals who had access to a personal glucometer was shown in another study [56]. In the study by Zhu et al. [57], a group of individuals with type 2 diabetes received additional care from their physician. intervention consisted of multiple sessions providing education on self-care, health counseling, and medication reminders in addition to standard diabetes care. Similar to the findings of Cengiz and Korkmaz [40], this approach had a positive effect on adherence to self-care within the intervention group. Moreover, the weekly SMS reminders, sent twice per week, had a significant positive impact on participants’ medication adherence [51].

### 3.4. Psychological Factors

#### 3.4.1. Motivation and Self Regulatory Factors

The results identify motivation as a central psychological factor influencing adherence to self-care [41,46,49,52,55]. One study distinguishes four levels of motivational mindsets—avoidant, disengaged, striving, and activated, which differ in willingness, goal orientation, and readiness for action. The avoidant and disengaged mindsets were associated with poor adherence [55]. An avoidant mindset involved evading self-care by avoiding thoughts about the disease or postponing self-care due to fear or shame, leading to low adherence. A disengaged mindset was characterized by indifference and low prioritization of health; diabetes was perceived as unimportant in daily life, resulting in inconsistent or absent adherence. This aligns with Endale et al. [41], who found that lack of interest was one of the most common reasons for poor adherence to physical activity. Individuals with a striving mindset wished to engage in self-care but encountered obstacles or struggled with persistence, requiring support and setting to maintain adherence. Finally, those with an activated mindset demonstrated high motivation and self-regulation, resulting in strong and consistent adherence to self-care behaviors [55]. Self-efficacy and perceived self-control were strongly correlated with greater adherence to self-care [49,55,56]. Stigma and self-blame undermined motivation and led to avoidance of support, thereby reducing adherence, whereas self-efficacy, clear intentions, and both short- and long-term goals enhanced it. Motivation operated on two levels: long-term goals (e.g., avoiding complications) and short-term goals (daily rewards, intermediate milestones, and immediate feedback) [55]. Motivation decreased when competing priorities, lack of knowledge, or lack of immediate rewards were present [46,55]. Individuals who perceived self-care activities as meaningful or rewarding were more likely to maintain motivation and adherence [52].

#### 3.4.2. Personal Characteristics

The findings indicate that personal characteristics influence adherence to self-care among individuals with type 2 diabetes [45,46,48,49,55,56]. Personal attributes, including specific personality traits, influenced adherence in varying ways [46,54]. Conscientiousness reflected in careful planning, adherence to routines, monitoring, and openness to trying new strategies was positively associated with adherence. Neuroticism, defined as a tendency to experience negative emotions such as anxiety, anger, and sadness, was linked to poorer adherence. Finally, a negative attitude toward one’s illness was associated with reduced adherence [45].

#### 3.4.3. Emotions, Mental Health and Psychological Distress

**Figure 2:**
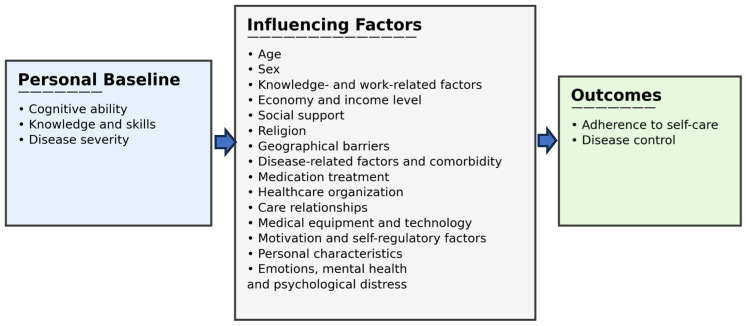
Visualization of the result.

The role of mental health in relation to adherence to self-care is addressed [41,42,45,48,49,50,54,55,56]. Emotions of self-blame and shame, particularly among younger adults (18–39 years), were associated with increased psychological burden and reduced adherence [49,55,56]. Shame, frustration, poor communication, and self-blame also contributed to withdrawal and diminished communication with the healthcare team [48,55]. Depression is common in chronic illness and is associated with poorer adherence to self-care, as depressive symptoms reduce energy, interest, and initiative [56]. A study from Saudi Arabia found that depression is not uncommon among individuals with diabetes, and that greater depressive severity was associated with poorer adherence [50]. Similarly, Lins Oliveira Frazão et al. [45] observed that the more depressed a person was, the less self-care they performed [45]. Anxiety impaired concentration and self-care planning, while anger reduced the likelihood of seeking support for self-management [42,48,50]. Diabetes-related stress, sadness, and fear were also described as factors that diminished motivation, energy, and the ability to plan daily self-care [48,49,55]. Mental fatigue was identified as a barrier to self-care, leading to concentration difficulties and reduced initiative. The study further indicated that fear of illness could result in avoidance of self-care [55]. Forgetfulness also contributed to lapses in self-care; when self-care was neglected due to forgetfulness, it could not be compensated for afterwards [54]. Despite challenges related to mental health and forgetfulness, some routines such as medication intake could still be maintained [45,54] (Figure 2).

## 4. Discussion

Main findings demonstrate that adherence to self-care is shaped by a wide array of interrelated factors. These include sociodemographic, healthcare-related, and disease-related factors, as well as social, psychological, and knowledge-related determinants. The results underscore that some factors are modifiable, whereas others are more predetermined and non-modifiable. This is comparable to the analysis presented by the Public Health Agency of Sweden [60], concerning the determinants that influence health and life opportunities, i.e., the determinants of health. According to Dahlgren and Whitehead [61], non-modifiable factors such as age and sex occupy the center. The model posits interactions across layers, implying that determinants influence each other [61]. This reasoning aligns with our results, which show that multiple factors are mutually involved and may affect one another. For example, financial strain may contribute to low mood and anxiety, which can undermine motivation and thereby reduce adherence to self-care. Individuals bear responsibility for their health and actions; favorable conditions increase the likelihood of achieving health, whereas limited opportunities reduce it though not inevitably resulting in poorer health [61]. This can be understood through the lens of self-care maintenance in Riegel’s theory, which emphasizes that self-care is influenced by personal resources, capabilities, and motivation, as well as environmental and situational conditions. The theory highlights that certain factors, such as knowledge and motivation, can be strengthened through targeted support, whereas others such as age or chronic illness are more stable and difficult to change. This perspective clarifies why some barriers to self-care can be addressed directly, while others require adaptation rather than alteration.

Our findings align closely with Riegel’s Self-Care Theory, which conceptualizes self-care as a process influenced by an individual’s personal baseline, a wide range of influencing factors, and their impact on self-care adherence and disease control. The model emphasizes that self-care is shaped by psychological, social, economic, and contextual conditions [31,35,36].

Prior research supports these findings, namely that self-care in type 2 diabetes is influenced by multiple circumstances [34,62,63]. Type 2 diabetes demands substantial self-care activities and that a range of personal and contextual factors affect adherence [34,63]. Living with type 2 diabetes is a challenge requiring a systematic, integrated strategy to facilitate self-care and strengthen adherence. Such strategies must account for patient-related and healthcare-related factors, consistent with the determinants identified in this review. Similarly, the considerable self-care burden of type 2 diabetes and the personal engagement required to achieve effective self-management. The studies also report that a substantial proportion of individuals with type 2 diabetes exhibit suboptimal self-care [34,63]. According to WHO [64] increases the risk of complications, with effects on service utilization, healthcare costs, and human suffering. Considering these, there is a strong rationale for optimizing and ensuring the quality of diabetes care from both societal and individual perspectives. The present findings are valuable from both perspectives. Managing public resources responsibly while reducing suffering and safeguarding quality of life is fully aligned with the 2030 Agenda and the Sustainable Development Goals [65].

The results highlight the decisive role of psychological factors in adherence to self-care in type 2 diabetes. Depression, stress, shame, and low motivation emerge as central barriers that can impede the maintenance of healthy routines even when knowledge and resources are present. Conversely, self-efficacy and emotional states are crucial for adherence. The findings also show that self-blame contributes to avoidance and impaired communication with the care team, thereby undermining adherence. These observations align with prior research demonstrating strong links between diabetes-related stress, psychological well-being, and self-care [66]. Self-efficacy functions as a protective factor by mitigating adverse psychological effects and fostering resilience in the capacity to adapt. Ataya et al. [67] report that self-efficacy often interacts with psychological factors in predicting adherence; individuals with high self-efficacy can maintain self-care despite mental health challenges and diabetes-related anxiety. Similar associations are reported where health literacy and self-efficacy were strongly associated with adherence. The study further underscores self-efficacy as a bridge between knowledge and self-care, emphasizing that psychological factors are essential for translating knowledge into action [68]. A recent systematic review has demonstrated that several factors facilitate effective diabetes self-management. These facilitators include strong motivation to engage in self-care, a positive attitude toward diabetes management and adequate knowledge of the disease and its treatment, as well as practical skills and self-efficacy that enable individuals to confidently perform diabetes-related behaviors. In contrast, commonly reported barriers include psychological challenges such as depression, along with practical obstacles such as polypharmacy or the complexity of medication regimens, which can make day-to-day management more difficult, which is in line with our study [69]. Person-centred care grounded in individuals’ needs, values, and circumstances, and inclusive of emotional support and lifestyle change appears central to strengthening capacity for self-care [68,70]. Without investments in psychological support, inequalities in care risk being exacerbated, particularly as individuals with lower socioeconomic status often have reduced access to psychological resources and support networks. Thus, psychological determinants are not solely individual but also reflect structural and societal differences. Motivation, self-efficacy, and emotional states are key components enabling effective self-care. When mental ill-health, fear, or shame are present, the capacity to perform these processes diminishes, not necessarily due to lack of motivation, but due to reduced ability [31,35].

From an ethical standpoint, the question becomes the extent of healthcare’s responsibility to support individuals’ capacity for self-care, rather than assigning blame. When psychological barriers such as depression, shame, or low motivation impede adherence, these insights should be used to enhance autonomy and participation, rather than to moralize non-adherence. Beaudin et al. [71] show that district nurses’ ability to tailor support and follow-up to individual needs is central to fostering engagement and motivation especially when mental health challenges compromise independent health management. District nurses should be mindful of how self-care is structured, as this can promote motivation and engagement [71]. The motivation to self-care must also be understood in relation to the right to autonomy. Autonomy concerns not only the making of decisions but also the conditions for making them; depression, anxiety, and shame can diminish these conditions [31]. The ethical responsibility of the district nurse is therefore to identify such barriers and actively support the person’s capacity for self-care, not merely to deliver information.

Personal traits such as conscientiousness and openness may support effective self-care. However, care models that assume a high level of self-discipline risk disadvantaging individuals with mental ill-health or low motivation, as their symptoms may reduce their ability. Individuals living with diabetes encounter several factors that influence their ability to perform effective self-management. According to Adu et al. [72], key enablers of successful diabetes self-care include the motivation to prevent long-term complications and the use of technological tools such as digital devices that support daily monitoring. At the same time, patients experience substantial barriers that complicate their self-management efforts. These barriers include frustration with the chronic and fluctuating nature of the condition, financial constraints, and unrealistic expectations regarding treatment outcomes. Additional obstacles relate to work and environmental circumstances, which may limit a person’s capacity to maintain consistent self-care routines. The study further highlights that educational support delivered through digital technologies, such as mobile applications, has the potential to strengthen self-management skills. They argue that interventions aimed at reducing financial burden, addressing work- and environment-related challenges, and alleviating diabetes-related distress are essential for improving the overall conditions for self-management, shaping realistic expectations, and enhancing the daily management of the disease [72]. A study focusing on individuals with type 2 diabetes and co-occurring mental illness shows that they frequently encounter distinct self-care challenges, particularly regarding their perceived ability to manage the condition. These findings highlight the need for individualized support to ensure ethically sound and equitable care [73]. Mental ill-health and emotional barriers strongly influence the capacity to translate knowledge into practical self-care. An observational study found that psychological well-being shapes both self-care practices and the application of knowledge. In this sense, psychological well-being can be viewed as a crucial link between knowledge and adherence [74].

Knowledge emerges as a foundational prerequisite for self-care but is not, by itself, decisive for adherence. Higher levels of knowledge do not automatically lead to improved routines; rather, the effect of knowledge is mediated by self-efficacy and supported by resources, care organization, and social support. This is in line with earlier research which found that knowledge is necessary but insufficient; health literacy, motivation, support, and experiential learning are critical for strengthening confidence and sustaining adherence [75,76]. Knowledge is required to perform self-care, monitor symptoms, and make management decisions. To translate knowledge into practice, individuals must not only know what to do but also be able to monitor health, remain motivated, and act on changes. Knowledge transfer should be practiced experientially to support decision-making and strategies that reinforce self-care [31,35]. Consistent structured nurse-led, digital, and practice-oriented interventions can enhance both knowledge and self-efficacy [77]. Combined structured Diabetes Self-Management Education with self-regulation-based and family-oriented components has been developed to achieve greater and more sustainable effects to design more person-centered educational programs [78]. Artificial intelligence has emerged as an effective self-care tool for individuals with diabetes mellitus, supported using several different technological models. Overall, artificial intelligence appears to enhance the conditions necessary for effective patient self-care [79].

## 5. Limitations

The review is based on a limited number of databases, which may have resulted in the exclusion of relevant studies published in journals not indexed in these sources. Including only studies written in English may introduce language bias and lead to the omission of valuable research published in other languages. Literature consists of studies employing heterogeneous methodologies, which makes consistent quality assessment challenging and potentially uneven. The literature search reflects studies available up to a specific point in time, meaning that research published after the cut-off date is not included. Nonetheless, the studies incorporated into the review generally represent research conducted in recent years and therefore reflect the contemporary state of knowledge within the field. The currency of the conclusions is assessed by evaluating the extent to which the findings align with and represent the current state of research.

## 6. Clinical Implications

For clinical implications, it is essential that district nurses working in diabetes care are familiar with the relevant health determinants. Both the present and previous research show that multiple factors are important for self-care adherence and health outcomes in type 2 diabetes. These factors should be integrated into diabetes care as part of promoting healthy lives and well-being. While non-modifiable factors must be acknowledged, attention should concentrate on those modifiable determinants that are specific to diabetes. Even though heredity and sex cannot be altered, it remains important to recognize that they may affect adherence. Understanding the complexity of influences on adherence is crucial for providing care and support consistent with science and proven experience.

## 7. Conclusions

Together, the present findings point to the same conclusion: improved adherence is achieved through integrated, personalized measures in which knowledge transfer is combined with training in practical skills, support for self-care decision-making, and actions that strengthen self-efficacy while reducing practical barriers. The capacity to translate knowledge into action is central not only to individual health and well-being but also to global goals of equitable access to knowledge, education, and health support. The overall findings indicate that adherence to self-care in type 2 diabetes cannot be understood solely as a result of an individual’s knowledge or motivation. Rather, it is a dynamic process in which psychological, social, economic, and organizational factors interact. The results show that a wide range of factors affect self-care adherence. Sociodemographic and psychological factors to disease-related and healthcare-related factors can play an important role in a person’s ability to adhere to self-care recommendations.

Patient education in self-care is a central component of traditional diabetes care, with a primary focus on providing knowledge about diabetes and its treatment. The findings suggest that while knowledge is important, knowledge alone does not always lead to improved adherence. They also indicate that the structure and organization of healthcare play a significant role in adherence, and that offering individually tailored care as a complement to traditional diabetes care is effective, especially when psychological factors are incorporated and taken into consideration. Adherence is strengthened when self-care is seen as meaningful, manageable, and socially supported. Type 2 diabetes requires substantial engagement from individuals, as the treatment involves multiple components such as medication, monitoring, and lifestyle modifications. The findings suggest that regardless of the healthcare approach used, care should be individualized and take relevant influencing factors into account, while also making use of each person’s own abilities. Supporting individuals with type 2 diabetes is a multidimensional task consisting of several components, aimed at providing the opportunity to achieve quality of life, metabolic control, and the prevention of complications.

## Figures and Tables

**Table 1:** PEO model.

PEO Model	Description	Search Terms
**P**opulation	Patients with type 2 diabetes	“type 2 diabetes”, “diabetes type 2”, “diabetes mellitus type 2”
**E**xposure	Self-care	“self care”, “self administration”, “activites in daily living”
**O**utcome	Adherence	Compliance, adherence, “patient * compliance”, “treatment adherence”

Note: * In information searching, truncation means shortening a word and using a symbol (most often an asterisk, *) to retrieve multiple word forms based on the same word stem.

**Table 3:** Example of the analysis process.

Meaning Units	Subcategories	Categories
Unemployed patients had significantly higher self-care scores than those who were employed.	Unemployed	Healthcare-related factors
Improved interaction between patients and healthcare professionals to resolve doubts/questions can enhance adherence.	Care relationship	Disease- and treatment-related factors
Diabetes-related complications and other comorbid conditions acted as barriers to participating in daily physical exercise	Complications	Disease-related factors and comorbidity
Since I live alone, I feel a bit down	Depression	Psychological factors

**Table 4:** Categories and Subcategories.

Categories	Subcategories
Sociodemographic factors	Age Sex Knowledge- and work-related factors Economy and income level Social support Religion Geographical barriers
Disease- and treatment-related factors	Disease-related factors and comorbidity Medication treatment
Healthcare-related factors	Healthcare organization Care relationships Medical equipment and technology
Psychological factors	Motivation and self-regulatory factors Personal characteristics Emotions, Mental health and psychological distress

## Data Availability

No new data were created or analyzed in this study. Data sharing is not applicable to this article.

## Supplementary Materials

The following supporting information can be downloaded at https://www.mdpi.com/article/10.3390/healthcare14070941/s1, Supplementary Materials S1: Search string; Supplementary Materials S2: Mixed Method Appraisal Tool (MMAT).

## Abbreviations

The following abbreviations are used in this manuscript:

MMAT	Mixed Method Appraisal Tool
SBU	Swedish Agency for Health Technology Assessment and Assessment of Social Services
