# Supplementary material for: Factors Influencing Adherence to Self-Care in Patients with Type 2 Diabetes: A Systematic Literature Review
Source: Healthcare (Basel). 2026 Apr 3;14(7):941. doi: 10.3390/healthcare14070941 (PMC13073129; doi:10.3390/healthcare14070941)
Supplement: Supplementary file 1 [file healthcare-14-00941-s001.zip › Supplementary Materials S1.pdf]

Supplementary Materials S1.

Search action	CINAHL	PsycInfo	PubMed
″ Diabetes mellitus type 2″ OR ″Type 2 diabetes″ OR ″Diabetes type 2″ OR ″Diabetes mellitus type 2″	84386	9739	248682
“Self-care” OR “Care, self” OR “Self care” OR “Self administration” OR “Activit* of daily living”	72182	38570	107120
“Patient compliance” OR “Treatment compliance” OR “Treatment adherence and compliance” OR Compliance OR Adherence OR ″Treatment adherence″	119210	68302	482667
#1 AND #2 AND #3	595	247	1139
Filter: English language	136	19	284
Limitation 3 years (2022-2025)	100	19	211

CINAHL

(MM "Diabetes Mellitus, Type 2" OR TX "type 2 diabetes" OR TX "diabetes type 2" OR TX "diabetes mellitus type 2") AND (MM "Self Care" OR MM "Care, Self" OR TX "self care" OR TX "self administration" OR TX activit* of daily living) AND (MM "Patient Compliance" OR MM "Treatment Adherence and Compliance" OR TX compliance OR TX adherence OR TX "treatment adherence")

Pubmed

("Diabetes Mellitus, Type 2"[Majr] OR "type 2 diabetes"[tiab] OR "diabetes type 2"[tiab] OR "diabetes mellitus type 2"[tiab]) AND ("Self Care"[Majr] OR "self care"[tiab] OR "self administration"[tiab] OR activit*[tiab] of daily living) AND ("Treatment Adherence and Compliance"[Majr] OR compliance[tiab] OR adherence[tiab] OR "treatment adherence"[tiab])

PsycInfo

(DE "Diabetes Mellitus Type 2" OR TI("type 2 diabetes" OR "diabetes type 2" OR "diabetes mellitus type 2") OR AB("type 2 diabetes" OR "diabetes type 2" OR "diabetes mellitus type 2")) AND (DE "Self Care" OR TI("self care" OR "self administration" OR activit* of daily living) OR AB("self care" OR "self

administration" OR activit* of daily living)) AND (DE "Treatment Compliance" OR TI(compliance OR adherence OR "treatment adherence") OR AB(compliance OR adherence OR "treatment adherence"))
