# Supplementary material for: Factors Influencing Adherence to Self-Care in Patients with Type 2 Diabetes: A Systematic Literature Review
Source: Healthcare (Basel). 2026 Apr 3;14(7):941. doi: 10.3390/healthcare14070941 (PMC13073129; doi:10.3390/healthcare14070941)
Supplement: Supplementary file 1 [file healthcare-14-00941-s001.zip › Supplementary Materials S2.pdf]

Supplementary Materials S2. Mixed Methods Appraisal Tool (MMAT), version 2018

Screening questions (for all types)	S1. Are there clear research questions?			S2. Do the collected data allow to address the research questions?		
	Yes	No	Cannot tell	Yes	No	Cannot tell

Screening questions (qualitative)	1.1. Is the qualitative approach appropriate to answer the research question?	1.2. Are the qualitative data collection methods adequate to address the research question?	1.3. Are the findings adequately derived from the data?	1.4. Is the interpretation of results sufficiently substantiated by data?	1.5. Is there coherence between qualitative data sources, collection, analysis and interpretation?	Quality Appraisal
Screening questions (Quantitative randomized controlled trials)	2.1. Is randomization appropriately performed?	2.2. Are the groups comparable at baseline?	2.3. Are there complete outcome data?	2.4. Are outcome assessors blinded to the intervention provided?	2.5 Did the participants adhere to the assigned intervention?	Quality appraisal
Screening questions (Quantitative non-randomized)	3.1. Are the participants representative of target population?	3.2. Are measurements appropriate regarding both the outcome and intervention?	3.3. Are there complete outcome data?	3.4. Are the confounders accounted for in the design and analysis?	3.5 During the studyperiod, is the intervention administered (or	Quality appraisal

					exposure occurred) as intended ?	

Screening questions (Quantitative descriptive)	4.1. Is the sampling strategy relevant to address the research question?	4.2. Is the sample representative of the target population?	4.3. Are the measurements appropriate?	4.4. Is the risk of nonresponse bias low?	4.5. Is the statistical analysis appropriate to answer the research question?	Quality appraisal
Screening questions (Mixed methods)	5.1. Is there an adequate rationale for using a mixed methods design to address the research question?	5.2. Are the different components of the study effectively integrated to answer the research question?	5.3. Are the outputs of the integration of qualitative and quantitative components adequately interpreted?	5.4. Are divergences and inconsistencies between quantitative and qualitative results adequately addressed?	5.5. Do the different components of the study adhere to the quality criteria if each tradition of the methods involved?	Quality appraisal
